# Genetic testing for channelopathies, more than ten years progress and remaining challenges

**DOI:** 10.4103/0975-3583.64429

**Published:** 2010

**Authors:** Peng Zhou, Junhua Wang

**Affiliations:** *Section on Cardiology, Internal Medicine, Wake Forest University School of Medicine, Medical Center Boulevard, Winston-Salem, North Carolina 27157, USA*; 1 *Department of Cardiology, Air Force General Hospital, PLA, No.30, Fucheng Road, Haidian District, Beijing 100142, China, PRC*

Cardiac channelopathy, or primary cardiac electrical disease indicates myocyte ion channel dysfunction due to encoding ion channel gene and related gene mutation. Channelopathy usually causes the unstable cardiac electrical activity and results in arrhythmia. Brugada syndrome, long QT syndrome and short QT syndrome are three paradigms of congenital cardiac channelopathies in which a single gene mutation causes clinical arrhythmia, syncope and sudden cardiac death (SCD). They are currently the best available channelopathy models for evaluating the relationship between genotype-phenotype and understanding the electrophysiological mechanisms for malignant arrhythmia. They also represent bridges between modern molecular biology and clinical cardiology. However, there are incomplete penetrance and substantial heterogeneity in genotype-phenotype relationships, resulting in a very broad clinical disease spectrum for each channelopathy. This heterogeneity can be manifested as carriers of silent gene mutations, different responses to drug challenge tests, asymptomatic individuals with spontaneous electrocardiogram (ECG) abnormalities, iterative syncope patients, and aborted SCD patients to SCD victims.[1,2] Identification of all disease-causing genes and the associated mutations will improve pre-symptomatic diagnosis and enable early intervention and follow-up of asymptomatic patients, although clinical data presently available show that genetic testing results cannot be used for prognostic forecast and risk stratification for Brugada syndrome.[3]

## DISCOVERY OF CAUSATIVE GENE MUTATIONS

Since Wang *et al*, reported genomic organization of the human *SCN5A* gene encoding the cardiac sodium channel in 1996,[4] the identification of mutations and investigation of genotype-phenotype relationships of channelopathies have become focal points in the field of genetics and cardiology. For Brugada syndrome alone, 7 related genes, and hundreds of associated mutations have been identified.[5,6] *SCN5A*, encoding the Nav1.5 α-subunit, causes the sodium ion channel "loss-of-function";[7] *GPD1L*, encoding glycerol-3-phosphate dehydrogenase-1 like protein, and whose mutations interact with sodium channel α-subunit, also leads to sodium channel "loss-of-function";[8] *CACNA1C*, encoding the Cav1.2 α-subunit, gives rise to calcium channel "loss-of-function";[9] *CACNB2*, encoding the Cav1.2 β-subunit, results in calcium channel "loss-of-function";[9] *SCN1B*, encoding the Nav1.5 β-subunit, induces sodium channel "loss-of-function";[10] *SCN3B*, encoding Nav1.5 β-subunit, induces sodium channel "loss-of-function";[11] *KCNE3*, encoding the β-subunit of several potassium channels, including Kv4.3, which conducts transient outward potassium current (*Ito*), brings on "gain-of-function" of slowly activated delayed rectifier potassium current (*Iks*) and *Ito*.[11] Mutations in *SCN5A* account for roughly 20%-30% of all cases of Brugada syndrome while mutations in the other 6 genes account for only a very small number of Brugada syndrome phenotypes. The remaining 70%-80% of patients who meet the clinical diagnosis criterion do not harbor any of the associated mutations.[6]

Interestingly, almost all mutations lead to "loss-of-function" in sodium channels or calcium channels, except for *KCNE3*, whose mutation gives rise to potassium channel "gain-of-function".[12]

Other exciting discoveries in the channelopathy field are (1) from silent mutant gene carriers to SCD, one mutation can cause distinct phenotypes; (2) different mutations of one mutant gene can result in various types of channelopathies; and (3) the combination of different mutations can lead to mixed phenotypes or "overlap syndrome". For example, *SCN5A* gene, if its mutation gives rise to sodium channel "loss-of-function", results in Brugada syndrome, family progressive cardiac conduction disease and sick sinus syndrome; if its allele mutations lead to sodium channel "delayed inactivation", it causes long QT syndrome type 3 (LQT3) clinical phenotype. Moreover, *SCN5A* mutant gene is also responsible for dilated cardiomyopathy, atrial fibrillation, and sudden infant death syndrome.[5,13] If more than one mutations of *SCN5A* co-exist, mixed or "overlap syndrome" clinical phenotypes may occur.[13] Hence, identifying causative genes and understanding the basis for ion channel functional abnormalities as well as the genotype-phenotype relationship is critical for explaining clinical phenomena and formulating appropriate therapeutic strategies. Clinically, Brugada syndrome and LQT3 possess distinct ECG phenotypes: right precordial ECG leads to V_1_ to V_3_ ST segment elevation for Brugada syndrome while QT interval prolongation for LQT3. However, these 2 clinical entities share some common clinical characteristics and presentations: including predominance in males; deadly arrhythmia events occurring more frequently at night or at rest; and no benefit or even harmful effect of β-blocker medication has. Therefore, in the viewpoint of the mutation, ion channel functional abnormality and genotype-phenotype relationship, we can find that all these phenomena are reasonable and β-blocker should be avoided for these patients.

The exciting achievements in research noted above have greatly enriched our knowledge of the electrophysiological basis of malignant arrhythmia. However, some significant problems have arisen in the field of channelopathy, as will be discussed below.

## WHAT ARE THE REMAINING CAUSES OF DISEASE FOR CHANNELOPATHY PATIENTS?

As noted above, among all of the Brugada syndrome causative genes, *SCN5A*, which includes hundreds of associated mutations, accounts for about 20%-30% of the Brugada syndrome clinical phenotypes, while mutations in the other 6 recently identified related mutant genes only account for one family or a very small number of Brugada syndrome phenotypes. What are the causes for the disease in the remaining 70%-80% of all patients? This is still a conundrum and a big challenge for researchers.[3,14]

## DEEP DILEMMA FOR INTERPRETATION OF THE GENETIC TESTING RESULTS OF CHANNELOPATHY

For asymptomatic long QT syndrome, Brugada syndrome and short QT syndrome patient family members, silent mutant gene carriers, positive drug challenge test patients and asymptomatic individuals with spontaneous ECG abnormality, accurate diagnosis is of critical importance. Theoretically, genetic testing is the "gold standard" to determine the preventive and follow-up plan, therapeutic strategy and prognostic estimation for these patients. However, for example Brugada syndrome, restricted by limited clinical and genetic data, cardiologists are usually confronted with the following challenges: (1) for 70%-80% of Brugada syndrome patients, the genetic test results are negative;[6] (2) even if the genetic test results are positive, the mutations may be harmless nonsense mutations;[14] 3) mutations may be harbored in all of us. For example, the *SCN5A* mutant gene is mutated in 2-5% of "normal" individuals although the associated mutations of Brugada syndrome are usually found in the seven transmembrane domains and pore-forming segments while the mutations of "normal" individuals are often located in linking areas;[6,14] (4) for most Brugada syndrome patients with identified mutations, the mutations are usually "private",[6] so it's impossible to use mutant function studies for every patient; (5) the *in vitro* mutant function study results may be different from the pathophysiological condition *in vivo*.[14] Practically, these are the toughest conundrums to cardiologists. Once the channelopathy diagnoses are established, patients and their families would endure tremendous psychological and social suffering; conversely, if diagnoses are overlooked, each arrhythmic event could be deadly. Faced with relatively young individuals who could be at risk, which course of action is appropriate for the cardiologist? This is a deep dilemma indeed.

## DRUG-INDUCED CHANNELOPATHIES

Some drug-induced channelopathies also represent a clinical dilemma.[15,16] First, little is known about whether there is existence of proarrhythmic substrates in the population with drug-induced channelopathies such as Brugada syndrome, due to limited randomized, double-blinded, controlled, long-term follow-up data and genetic data from international multi-center collaborative clinical trials. Second, it is not clear whether these patients always lie in cardio-electrical stable condition, and if there is a genetic predisposition in these patients. Fortunately, 10%-15% of drug-induced QT-prolongation individuals who developed *tosades de points* (TdP) possess mutations associated with long QT syndrome.[17] This result perhaps provides us with an important clue to handle the population of drug-induced Brugada-like ECG patterns.

Therefore, in the future, answers to overcome these problems raised above will probably rely on: (1) randomized, controlled, international multi-center collaborative clinical trials, and long-term follow-up accumulative data; (2) identification of more disease-causing genes and mutant sites, as well as reinforcement of mutant function studies for enhancement of our genetic database incessantly.

In this issue, Dr. Liang et al. report 2 novel mutations in the *SCN5A* gene. 1 of the mutations is associated with Brugada syndrome and the other with LQT3, based on genetic analysis of 4 diagnosed and 9 suspected Brugada syndrome patients and 3 LQT3 Chinese patients.

These results enrich the channelopathy genetic database although further mutation-function studies are necessary to confirm the physiological relevance of these findings.
